# A Review of the Current Mammalian Models of Alzheimer’s Disease and Challenges That Need to Be Overcome

**DOI:** 10.3390/ijms222313168

**Published:** 2021-12-06

**Authors:** Natasha Elizabeth Mckean, Renee Robyn Handley, Russell Grant Snell

**Affiliations:** 1 Applied Translational Genetics Group, School of Biological Sciences, University of Auckland, 3a Symonds Street, Auckland 1010, New Zealand; nmck309@aucklanduni.ac.nz (N.E.M.); r.handley@auckland.ac.nz (R.R.H.); 2 Centre for Brain Research, Faculty of Medical and Health Sciences, University of Auckland, Auckland 1010, New Zealand

**Keywords:** Alzheimer’s disease, animal model, transgenesis, gene editing, large animal model, plaques, tangles, predictive validity, construct validity

## Abstract

Alzheimer’s disease (AD) is one of the looming health crises of the near future. Increasing lifespans and better medical treatment for other conditions mean that the prevalence of this disease is expected to triple by 2050. The impact of AD includes both the large toll on individuals and their families as well as a large financial cost to society. So far, we have no way to prevent, slow, or cure the disease. Current medications can only alleviate some of the symptoms temporarily. Many animal models of AD have been created, with the first transgenic mouse model in 1995. Mouse models have been beset by challenges, and no mouse model fully captures the symptomatology of AD without multiple genetic mutations and/or transgenes, some of which have never been implicated in human AD. Over 25 years later, many mouse models have been given an AD-like disease and then ‘cured’ in the lab, only for the treatments to fail in clinical trials. This review argues that small animal models are insufficient for modelling complex disorders such as AD. In order to find effective treatments for AD, we need to create large animal models with brains and lifespan that are closer to humans, and underlying genetics that already predispose them to AD-like phenotypes.

## 1. Introduction

Alzheimer’s disease (AD) is a devastating neurodegenerative disease, behaviourally characterised by memory loss and cognitive decline, generally in later life, which is ultimately fatal [1]. The prevalence of AD is rapidly increasing due to an ageing population worldwide, and expected to triple between the years 2000 and 2050 [2,3]. Besides those affected, AD places a severe burden on families, carers, and the economy [4,5]. Alois Alzheimer discovered the neuropathological hallmarks of AD in 1906 [6]. Despite many decades of research since the 1900s, a cure has remained elusive with current therapies only offering temporary symptomatic relief.

Classically, AD is characterised by plaques and tangles, both of which contain insoluble protein deposits that progressively accumulate in the brain [7,8,9]. These pathological features develop over decades, and considerable effort has been devoted to their replication in short lived models. Implicit in these modelling efforts is that the relatively rare dominant genetic forms of AD represent the condition as a whole, and that the accelerated processes artificially engineered into these models accurately represents the mechanisms of a slow disease process in humans. Most models have been constructed to recapitulate the end stage pathological features, assuming that they represent the cause of the condition rather than the consequence. To a large extent, this pathology attainment strategy for AD animal model construction has driven the preclinical selection of compounds going through to human clinical trials. Well over 200 compounds have now failed to prevent, slow, or cure the disease, despite most being effective at ‘curing’ mouse models of AD [10,11,12]. This review summarises the modelling efforts up to November 2021. It briefly covers the extensive work undertaken to capture AD symptomatology predominantly in the mouse, and summarises the key factors that still need to be obtained. Finally, suggestions are given for an alternative modelling strategy through the use of large animals.

## 2. The Defining Pathological Features of AD; Plaques and Tangles

**Figure 1:**
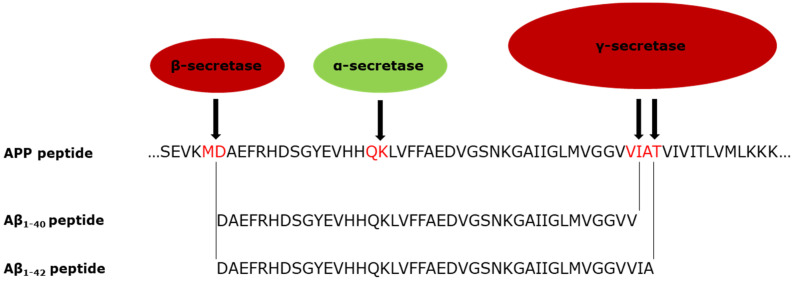
Cleavage of APP by beta and gamma secretase (in red) to release the Aβ_1__–__40_ and Aβ_1__–__42_ peptides associated with AD, and showing the alternative cleavage position of alpha secretase (green) [17,19].

Plaques are extracellular deposits formed from the amyloid-beta (Aβ) peptide; cleavage products of the transmembrane amyloid precursor protein (APP) [7,13,14,15,16]. APP is cleaved in two regions, releasing peptide fragments (Figure 1). It is cleaved by either an alpha or beta secretase enzyme in the lumen closer to the C terminus. It is cleaved nearer the N-terminus by the gamma secretase complex in a membrane domain. The gamma secretase complex can cleave at multiple sites within its target region of the APP protein, creating different sized products [17]. If APP is cleaved by alpha secretase, the peptides released are shorter and appear to be harmless. When APP is cleaved by a beta secretase instead, usually the BACE1 enzyme, longer (Aβ) peptides are released. Amyloid beta has the potential to aggregate into plaques. Aβ_1-__–__40_ and Aβ_1__–__42_ are the longest peptides and have the strongest association with AD, with Aβ_1__–__42_ deemed to have a causal role [18].

Tangles are dead or dying neurons containing intracellular aggregated hyper-phosphorylated TAU protein and other aggregated proteins [20]. In its native state, TAU is a structural component of axons within neurons, and it is unclear why it becomes hyper-phosphorylated [21]. The accumulation and density of tangles correlates better with neurological symptoms than plaques [22,23]. However, plaques begin forming before tangles, which led to the ‘amyloid cascade hypothesis’. The original amyloid cascade hypothesis proposed that the formation of Aβ plaques initiates the disease process and eventually leads to tangle formation, cognitive symptoms, and neurodegeneration [24]. It has subsequently been modified to suggest that an increase in the soluble toxic forms of amyloid beta, which precede plaque formation, initiates the disease process [25,26].

Cases of AD are broadly classified as early-onset Alzheimer’s disease (EOAD) or sporadic late-onset AD (LOAD). Some of the early onset cases can be further subdivided into a familial early onset group (FAD) caused by single genetic mutations, typically inherited in an autosomal dominant fashion [27]. LOAD is the most prevalent form of AD and is more complex, influenced by both genetic and environmental risk factors. The FAD patients (<5%) are of particular interest because the clear genetic aetiology offers an opportunity to understand the molecular mechanisms involved. It also allows the disease to be predicted before the onset of symptoms, and therefore mutation carriers are an ideal cohort for clinical testing [28]. Aside from age at onset, these two forms of AD (FAD and LOAD) are typically indistinguishable [29].

Over 200 mutations have been identified as responsible for FAD, all clustered into three genes; *APP*, Presenilin 1 (*PSEN1*), and Presenilin 2 (*PSEN2)*. As already introduced, the APP protein is cleaved to form Aβ. The *APP* gene is found on chromosome 21 and Trisomy 21 carriers (Down’s syndrome) have a high frequency of early onset AD, presumably caused by an increased dosage of APP [13]. The PSEN1 and PSEN2 proteins form part of the gamma secretase complex that cleaves the APP protein [30]. Mutations in *PSEN1* are the most common cause of FAD. Mutations in *PSEN2* are relatively infrequent and generally result in a later onset and slower disease course that those in PSEN1 (see review [31]). In summary, mechanistically it is thought that mutations in *APP*, *PSEN1*, and *PSEN2* favour the production of the more amyloidogenic, Aβ_1__–__42_ form of amyloid (reviewed elsewhere [32,33]).

Briefly, the risk of developing LOAD has been associated with variations in an increasing number of genes as the power of the genome wide association studies (GWAS) increases. Over 75 risk loci have now been detected [34], with twin studies revealing that up to 79% of the risk for LOAD is genetic [35]. The gene with the largest effect is Apolipoprotein E (*APOE*). The *APOE* gene has three variants (*APOE2*, *3*, and *4*). Inheritance of the *APOE2* allele is protective, the *APOE3* allele is a neutral effect on risk, and the *APOE4* allele confers a very significant risk for LOAD in a dose-dependent manner. *APOE4* heterozygotes have AD susceptibility an odds ratio of 3.5–4 and homozygotes 12–15 [36]. All of the genes that have so far been implicated in AD can be functionally linked to Aβ peptide homeostasis [37,38,39], implying a common mechanism that could shed light on the aetiology of the disease.

## 3. Modelling AD in Animals

One of the most effective ways of investigating the pathogenic process of a disease is via animal models. Animal models can also be used for biomarker discovery, which can allow for early detection of disease, and for screening and safety tolerance testing of therapeutic agents. There are three main aspects of animal modelling that need to be considered: the resulting face, construct, and predictive validities [40]. These relate to how well the model replicates symptoms, the biological causes, and responds to clinically effective therapeutics, respectively.

The earliest animal models of AD were created by disrupting the cholinergic system in various mammalian species using surgical methods, neurotoxins, immunotoxins, or pharmacological methods. The species targeted included mice and rats [41], rabbits [42], and monkeys such as the marmoset and crab eating macaque [43,44]. The cholinergic system in the basal forebrain degenerates early in the course of AD [45,46]. These models replicated some of the symptoms of AD such as memory impairments, and were helpful for testing the efficacy of cholinesterase inhibitors, which can offer some symptomatic relief early in the course of AD [41]. These models, of course, did not develop plaques or tangles, nor did they represent the progression of the complex biochemical and cellular-level changes in AD [47].

The rapid development of genetic technology and engineering from the 1980s to the present has enabled the construction of animal models that can theoretically recapitulate diseases from their underlying causes, thus increasing the construct validity of the model.

## 4. Small Animal Models of AD

### 4.1. Mouse Models

#### 4.1.1. Plaque Pathology in Mouse Models

As a mammalian model system, mice have the advantages of a short lifespan and rapid reproduction, which facilitates timely completion of experimental protocols. They are also comparatively easy to maintain and breed in a laboratory environment. Numerous tools, data, and standardised behavioural tests have been established for assessing phenotypes in mice. The development of embryonal stem cells and targeted mutagenesis has enabled the production of models that more accurately recapitulate the aetiology of human disease state. These factors combined has resulted in mice being the most common animal models of AD.

There have been a large number of mouse models constructed in various ways, far too many to include here. We have selected a representative group of models that were either notable because they were novel at the time or have been widely used in the field. Table 1 lists these selected mouse models.

Plaques and tangles are the two main pathological hallmarks of AD, followed by neurodegeneration. In order to create models with high face validity, these phenotypes have been highly sought after. The first reported mouse models that developed plaque pathology were created via transgenesis (TG). Researchers introduced the human *APP* gene (h*APP*) containing mutations known to cause FAD. The first mouse model, the PDAPP line created in 1995, overexpressed the V717F Indiana mutation h*APP* with the Platelet-Derived Growth Factor (PDGF) promoter via a minigene construct. Around 40 copies of the transgene were randomly inserted in this line at a single site, and all three major splice variants of h*APP* (695, 751, and 770) were expressed. These mice developed both dense and diffuse plaque pathology by eight months of age in the entorhinal cortex, cingulate cortex, and hippocampus. By 18 months, the amyloid burden in these brain regions was thought to be greater than that seen in end stage human disease. This model also showed signs of synaptic loss, microgliosis, and astrocytosis, but no tau/tangle pathology or neurodegeneration [48,58].

The next, and still commonly used mouse model, was the Tg2576 line, which overexpressed the K670M/N671L Swedish mutation in a transgene containing the 695 isoform of human h*APP* transgene driven by the Prion Protein (PrP) promoter. Tg2576 mice develop plaques and memory deficits in a progressive manner. Similar to the PDAPP mice, they do not show the tangles or neurodegeneration [58,59]. These mouse models developed memory deficits and synaptic loss preceding the accumulation of insoluble plaques, providing evidence for the hypothesis that it is the smaller soluble forms of Aβ that cause these symptoms [60,61]. Several further mouse lines were also created by introducing the h*APP* gene with various FAD causing mutations; most exhibited plaques and memory deficits in an age-dependant manner as well as some level of synaptotoxicity (reviewed in [62]).

Some of these mouse lines were subsequently crossed to produce mouse lines with multiple *APP* transgenes; the result was usually a similar phenotype that appeared at an earlier age, which shows that these mutations have cumulative phenotypic effects. One example is the TgCRND8 line, engineered with a single transgene to contain the h*APP* isoform 695 with both the Swedish and Indiana mutations under the control of the Prp promoter. These mice develop plaque pathology by three months of age, with earlier signs of cognitive impairment relative to the models with a transgene carrying a single AD mutation. The brain concentration of Aβ_1__–__42_ in this compound model at six months was equivalent to the original PDAPP mouse line at 16 months. This compound model also showed an increase in Aβ_1__–__42_ to Aβ_1__–__40_ ratio (now considered to be an important indication of amyloidogenesis) [50]. However, these mice still did not exhibit the other major neuropathological hallmarks of AD such as the tangles and neurodegeneration.

Some of the APP overexpression mouse lines were later crossed with mice carrying a human *PSEN1* transgene (h*PSEN1*) with various mutations responsible for FAD. Interestingly, mice overexpressing h*PSEN1* mutations do not develop plaques or other symptoms, but do exhibit an increased ratio of the more amyloidogenic Aβ_1__–__42_ relative to Aβ_1__–__40_ in the brain [63,64,65]. Crossing transgenic mice that overexpressed *APP* with *PSEN1* transgenic mice greatly increased amyloid pathology. An example is the crossing of Tg2576 mice (*APP* Swedish mutation) with both the PS-1 line (*PSEN1* M146L mutation) [51,65] and the *PSEN1* A246E line [63,66]. Taken together, this animal model work helped confirm that APP metabolism, and in particular, the production of the Aβ_1__–__42_ peptide, was affected by mutations in *APP* and *PSEN1*, and that these mutations are likely acting on a single pathway. This work also provided supporting evidence for the hypothesis that the Aβ_1__–__42_ fragment is more toxic than Aβ_1__–__40_.

Attempts to confirm the role of individual Aβ peptides led to the creation of transgenic mouse lines that selectively expressed either the Aβ_1__–__40_ or Aβ_1__–__42_ amyloid fragment in the absence of the h*APP* transgene (BRI-Aβ40 and BRI-Aβ42) [52]. These models showed that high expression of Aβ_1__–__40_ caused no overt plaque pathology, but even low expression levels of Aβ_1__–__42_ was sufficient to cause plaque formation in both parenchymal brain tissue and blood vessels (cerebral amyloid angiopathy).

Attempts to capture a more complete AD phenotype led to crossing transgenic mice or creating constructs to overexpress multiple transgenes and mutations within these genes. Cell loss and neurodegeneration was ultimately achieved in the 5XFAD mouse model that expressed three *APP* (Swedish K670M/N671L, Florida I716V, and London V717I) and two *PSEN1* (M146L and L286V) mutations under the murine Thy-1 promoter [53,67]. The severe phenotype again supported the hypothesis that FAD mutations have an additive effect. However tangles, which are the other main hallmark of AD, were absent in these mice.

#### 4.1.2. Replicating AD Tau Pathology

Interestingly, unlike other mammalian species (see below), wild type mice do not develop tangles as they age [68]. Mutations in the human *MAPT* gene (microtubule associated protein tau), which codes for the human TAU (hTAU) protein, cause frontotemporal dementia (FTD), but not AD [69]. However tangle pathology, neurodegeneration, and memory loss were seen in transgenic mice models expressing human *MAPT* (h*MAPT*) with FTD causing mutations. The first mouse model with this phenotype was the JNPL3 line, which expressed the 4R0N isoform of h*MAPT* with the P301L mutation [54]. Subsequently, a hTAU expression tetracycline repressible mouse line (rTg4510) demonstrated that the smaller soluble forms of oligomeric TAU caused memory loss and neurodegeneration [70,71]. Many overexpression h*MAPT* transgenic lines have been produced and some have been crossed with transgenic mouse lines overexpressing FAD mutations in *APP* and/or *PSEN1*. The resulting lines demonstrated that the mechanisms leading to amyloid and TAU pathology interact. The 3xTg mice (Swedish mutation in *APP*, M146V in *PSEN1*, and P301L in *MAPT*) develop plaques before tangles [71,72], as observed in AD patients. A line developed by crossing the aforementioned *APP* mutant mice Tg2576 with the *MAPT* JNPL3 mice (called the TAPP line) altered the spatial distribution of tangles in the brain relative to original TAU expressing strain, with TAPP mice exhibiting tangles in the subiculum, hippocampus, and isocortex that were not present in JNPL3 mice. TAPP mice also had greatly increased numbers of tangles in the olfactory cortex, entorhinal cortex, and amygdala. This suggests that Aβ fibril deposition can alter the amount and distribution of insoluble TAU as tangles [57].

#### 4.1.3. Construct Validity of Transgenic Mouse Models of AD

Although mice expressing a transgene with a single FAD mutation display some symptoms of the disease, it is evident from the literature that three or more AD and FTD associated mutations are required to replicate the majority of the human pathology. In contrast, multiple mutations have not been reported in humans with AD, and in nearly all cases of FAD, only a single mutation is required to develop the entire phenotype.

**Figure 2:**
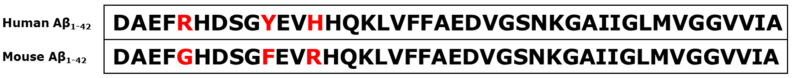
A comparison of the human and mouse Aβ peptide sequence, showing the three amino acid substitutions responsible for the functional difference between the two.

There are good reasons for the requirement of a compound approach to create equivalent AD pathology. Unlike human h*APP*, the proteolytic cleavage products of murine *App* (m*App*) do not naturally form plaques. This is due to three amino acid substitutions in the amyloid beta sequence compared to human (Figure 2), which reduces the ability of murine Aβ peptides to aggregate [72]. In addition, murine β-secretase enzymes typically cleave m*App* to form Aβ_11-x_, even though it cleaves h*APP* to form Aβ_1-x_ [73,74]. Deposition of cleavage products from m*App* is only apparent in models with high expression levels of m*App* and only after an extended period. This is one of the reasons why h*APP* is typically used instead [75].

The ratio of h*APP* isoforms differs within brain regions and also in other organs. The ratio also changes during the course of development and ageing [76,77]. The two longer isoforms of h*APP,* 751 and 770, are more prevalent in the AD brain relative to healthy controls [78]. Overexpressing the h*APP* 751 isoform also causes more obvious amyloid pathology in mice than overexpressing the short (APP695) isoform [79]. The pathology generated in a mouse model therefore depends on which of the three isoforms of h*APP* is overexpressed, or whether the full h*APP* gene sequence is used.

Several different promoters have been used to drive overexpression of h*APP* in mouse models of AD including the promoters for PDGF-B (platelet derived growth factor B-chain) and the PrP (prion protein gene motifs). Different promoters drive different levels and spatial patterns of expression including outside the brain. For example, the PDGF-B and Thy-1 (thymocyte differentiation antigen 1) promoters are neuron-specific [80,81], while the PrP promoter has less specificity, also driving expression in glial cells and other non-brain tissue [82]. The Thy-1 promoter included in the construct to make the APP23 model (Swedish mutation in *APP*) is active only after birth, preventing potential developmental effects [83]. Various Tet-controlled lines have been created that allow for more control over the timing and location of transgene expression, but have the added complication of requiring an extra transgene [84,85,86]. All of these promoters are selected for ease of use or particular benefits, but because none of them are the endogenous promoter, the natural expression pattern of *APP* is not replicated in any of the models.

#### 4.1.4. Murine APP Knock in Models

In an attempt to overcome the limitations of *APP* TG models, a small number of knock in (KI) *App* models have been created with targeted gene editing. Inserting selected mutations in the endogenous genes should mean expression is quantitatively, spatially, and temporally appropriate. Mouse *App* was ‘humanised’ in these models by converting the codons for the three amino acids that differ between human and mice in the Aβ coding portion of m*App*. This allows murine BACE1 to cleave mAPP at the human equivalent position [87,88,89,90]. These mice did not develop overt phenotypes such as memory deficits, synaptic loss, and/or plaque pathology. These phenotypes only became evident after the insertion of multiple *APP* mutations (combinations of Swedish, London, Dutch, Iberian, and Artic) [91], and usually only after breeding to homozygosity in concert with homozygous FAD *PSEN1* mutations [90,92].

The necessity of including multiple mutations to induce human equivalent disease confounds the use of these models, but they have helped differentiate between phenotypes due to the TG process, and those that represent the disease in a mouse. Consistent phenotypes observed in TG and KI models include plaque formation, changes to glial cells and astrocytes, and lowered rates of hippocampal neurogenesis, although some artifacts such as transgene calpain activation have been noted [93,94,95]. The presence of cognitive impairment appears to vary more between KI models than TG models. The KI models with cognitive impairment have plaque pathology prior to memory impairment, unlike the commonly used TG mice models [89,96]. Memory impairment following plaque formation is the order of events seen in patients [97], so KI models do appear to more faithfully replicate symptom clusters. Despite this, the higher variability of phenotypes in KI models, along with their milder symptom profile, means that transgenic models are still widely used.

#### 4.1.5. Murine PSEN1 Knock in Models

Murine *Psen1* (m*Psen1*) does not require ‘humanising’ like the m*App* and (m*Psen1*) models made with targeted gene editing by introducing FAD mutations, which show similar phenotypes to TG h*PSEN1* mouse lines [98,99]. Whether they are created by transgenesis or targeted gene editing, in the absence of h*APP* or humanised m*App*, all modelled *PSEN1* mutations only increased the level of murine Aβ_1__–__42_ in mice, had little or no effect on murine Aβ_1–40_ levels, and did not result in AD equivalent symptoms [65,100,101,102]. For this reason, more recently generated KI mouse models carrying a *PSEN1* mutation usually incorporate a transgene overexpressing mutant h*APP*. The resulting animals have a more acute phenotype than *APP* mutations alone [103,104,105].

#### 4.1.6. Construct Validity of MAPT Mouse Models

Compared to hTAU with six isoforms (named 4R2N, 4R1N, 4R0N, 3R2N, 3R1N, and 3R0N) [106,107,108] murine TAU (mTAU) only has three of the human equivalent isoforms (4R0N, 4R1N, 4R2N). There is also variability in TAU protein conservation. Some regions of mTAU tau are very similar to hTAU, while other regions differ greatly. There are species-specific differences in the presence of different isoforms during development, and spatially across the brain [108,109]. In TG models, the presence of endogenous mouse Mapt (m*Mapt)* can alter the splicing ratios of introduced h*MAPT* [110,111].

As stated above, unlike in humans, tangles do not form naturally with age in the mouse brain. Indeed, it appears that replacing the m*Mapt* gene with the human equivalent, and in some lines with a FTD mutation, is necessary to create a TAU dysfunction phenotype in mice [112]. The inclusion of FTD mutations to ensure a tangle phenotype in murine models is a major issue for construct validity. There are probably better models of frontotemporal dementia and other tauopathies than AD, even though they have provided insights about TAU toxicity [56,57]. Unexpected non-disease associated deficits have been found in some models, for example, the commonly used JNPL3 line (P103L mutation in *MAPT*) has motor impairments and develops eye irritations [54,113]. Further the Tau P301S line develops severe paraparesis at 5–6 months [114]. However severe motor impairment is not usually observed in AD until late in the disease course [115].

#### 4.1.7. Predictive Validity of Murine Models

Almost no mouse model of AD has shown predictive validity in human clinical trials to date, despite many therapeutic agents ‘curing’ a mouse of AD symptoms (for reviews, see [112,113]). Those that have been successful were based on the cholinergic system or NMDA receptors and only provide temporary symptomatic relief. While symptomatic relief is important, the predicted increase in the prevalence of AD means that finding a method to prevent or cure the disease is now becoming an urgent priority.

In addition to drug failures, there is the issue of differences in drug metabolism between species; something well tolerated in mice may not be so in humans [116,117]. Many clinical trials have failed to make it to later stages due to adverse side effects, which were not present in mice. For example, immunisation of mice with Aβ_1–__42_ (named AN1792 in the clinical trial) was able to lower the volume of plaque material in the brain and preserve cognitive function. Unfortunately, this approach failed to show benefits in clinical trials and 6% of the immunised patients developed meningoencephalitis [118,119]. The adverse effects were thought to be due to a T-cell response in humans against the large Aβ_1–__42_ fragment. Subsequent immunisation trials with smaller epitopes that were beneficial in mice including the drugs Bapineuzumab [120] and Solanezumab [121] showed a similar lack of efficacy and/or adverse side effects [122,123,124].

To date, well over 200 compounds have failed to affect the disease course [10], and this appears to have led to some controversial decisions. Recently, the drug aducanumab (sold as Aduhelm) was approved by the FDA through an accelerated approval pathway, on the condition that follow-up trials are performed to determine efficacy. This drug showed mixed results in clinical trials, with a benefit seen at the highest dose, but only in one of the two trials. Given that 35% of patients developed brain swelling (cerebral adema) and 19% brain bleeds (intracerebral haemorrhage), there are serious safety considerations [125]. It is clear that models of AD with higher predictive validity are desperately needed.

#### 4.1.8. Murine Model Summary

**Figure 3:**
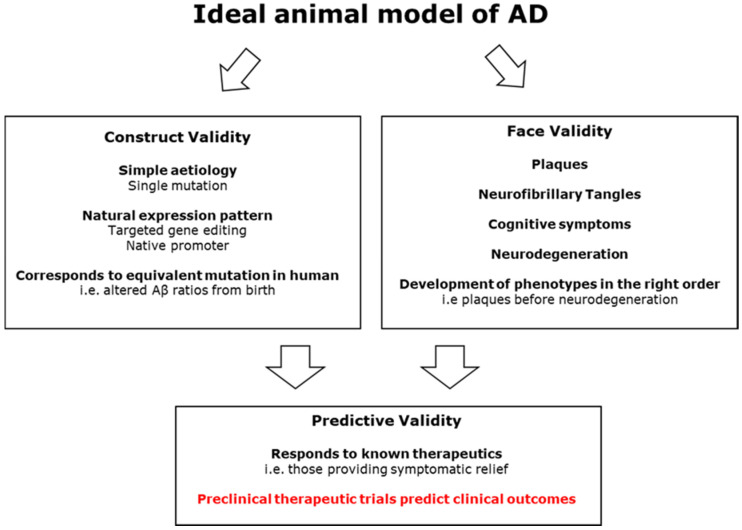
A diagram showing how a model of AD with high face and construct validity is likely to improve predictive validity and lead to effective therapies.

In summary, while successive generations of mouse models come closer to attaining the desired symptom clusters, this has created a trade-off between face and construct validity. The drive to replicated AD’s defining features of both plaques and tangles in a model is understandable. However, the inclusion of multiple mutations, with some from a different condition altogether, brings the construct validity of these models into question. Do they represent the disease process or a derived phenocopy? Discovering the mechanism by which amyloid pathology triggers TAU dysfunction would be invaluable for understanding the disease. Unfortunately, it appears that the mouse is too genetically and physiologically dissimilar to be able to capture this transition, even with genetic modification. The lack of translatability of treatments developed using these models is suggestive that they may not adequately represent AD. Work to understand the mechanistic nature of various FAD and MAPT mutations continues, and many of the aforementioned models are still utilized. Mouse models of LOAD variants have also been made including APOE and TREM2 [126,127,128]. It is likely that mechanistic work via mouse models will continue as more LOAD disease related genetic or environmental risk factors are discovered. However, over the last ten years, there have been repeated calls for new models of AD that can show predictive validity, causing researchers to look outside mice. Figure 3 summarises the desired qualities of an AD model, showing how improved construct validity could lead to higher translatability in clinical trials.

### 4.2. Rat Models of AD

Rats are an attractive model system because they are genetically and physiologically more similar to humans than mice. They display more complex behaviour, and numerous assessment methods have been developed for mood and cognition for this species [47,129,130,131]. The first rat models of AD were based on knowledge from mouse models, designed to express h*APP* with FAD mutations such as the UKUR25 line (with the Swedish and Indiana *APP* mutations, with the M146L *PSEN1* mutation [132]) or the McGill-R-Thy1-APP line (with the Swedish and Indiana h*APP* mutations, expressed under the murine Thy1.2 promoter [133,134]). Interestingly, these models failed to develop the plaques seen in mice, but did accumulate intracellular Aβ and developed memory deficits seen in the equivalent mouse models. Rat models that exhibited plaque pathology were finally created in the mid 2000s, sometimes with differing phenotypes from their murine genetic equivalents. The rat TgF344-AD line carries both Swedish h*APP* and *PSEN1* ΔE9 mutations, driven by the same murine PrP promoter [135]. This line develops both plaque and tangle-like pathology with loss of neurons. Interestingly the tangle-like structures appear despite the non-inclusion of a h*MAPT* transgene, even though tangles are not naturally seen in aged rats. This may be because unlike m*Tau*, rat *Tau* (r*Tau)* is spliced to create all six human equivalent isoforms [136,137]. Rat models expressing h*MAPT* with FTD mutations have also been developed, some of which exhibit tangles, while all show increased phospho-TAU in the brain and develop cognitive symptoms [138,139,140]. The development of Tau pathology, along with their larger brain and more complex behaviours, may confer on these models the potential to improve our understanding of AD.

## 5. Large Animal Models in AD Research

In pursuit of more translatable results across the medical sciences, researchers are more frequently turning to large animal models, particularly those that are evolutionarily closer to humans and have longer lifespans, thus making them better suited to recapitulate complex human diseases, especially late onset disorders [141,142,143]. Massively overexpressing transgenes speeds up the development of a phenotype, but also leads to acute inflammatory processes not present in human AD. Small mammals also have a smooth (lissencephalic) brain, while most larger mammals including humans have a more complex convoluted (gyrencephalic) brain. This makes larger mammals ideal for studying neurological disorders.

Many large animals naturally develop plaques and/or tangles as they age, and these features may be the norm in larger animals. They have been found in many primate species, and across a range of large herbivorous and carnivorous animals (summarised in Table 2). This propensity to develop plaques appears to be due, at least in part, to conservation of the amyloid beta peptide sequence in most mammals [144]. Of note is that in some large animals (e.g., dogs, sheep), only one of either plaques or tangles were originally identified, but later research revealed both [145,146]. They are typically found in aged animals, and vary widely in density between individuals of a species, so it is entirely possible that both hallmarks of AD will eventually be found in most large animals. Age related neurodegeneration has not been extensively studied in most species (Table 2), however, many are known to develop cognitive decline with age. Cognitive decline has rarely been studied in detail outside of humans, dogs, cats, and some primates.

Humans are fairly unique in outliving our reproductive lifespan, which no doubt contributes to the presence of AD and other dementias [191]. However, if other animals with medium to long lifespans can develop the hallmarks of AD, it is likely that the development can be accelerated with mutations from FAD. Large animals have long enough lifespans that introducing a single FAD mutation via KI methods will likely accelerate the disease in line with natural human forms of AD. This would remove the need for artificial promoters to massively overexpress transgenes to generate a robust phenotype within the 1–2 year lifespan of a mouse. If a large animal can develop all of the hallmarks of AD with the introduction of a single FAD mutation, this will represent a major step forward for construct validity. It would also be the first model to fully recapitulate a form of AD from its underlying cause.

### 5.1. Primate Models of AD

Being our closest relatives, primates show great promise for accurately representing human disease. Evidence of age related plaques, tangles, or both have been found across a range of primate species (see Table 2). Like humans, the pathological hallmark are only seen in some individuals, and is more likely with age, suggesting biological or environmental triggers that make certain individuals more prone to these precursors of dementia [147]. The great apes (bonobo, chimpanzee, orang-utan, and gorilla) have the highest genetic similarity to humans. Ethical concerns combined with slow reproduction make these animals relatively impractical models of AD [192,193,194]. Efforts have instead focussed on smaller primates already utilised to model human behaviour and disease.

Macaques are a promising species for modelling AD with a lifespan of 30 to 40 years, reaching old age around 20–25 years [183,195,196]. Elements of AD have been identified in multiple macaque species (Table 2). In particular, the rhesus monkey is a relevant model based on extensive data collected on the ageing process in these animals, and the similarity of their plaque morphology and staging to human [158,197,198].

Two smaller monkey species, the mouse lemur and the common marmoset, have also been considered for AD modelling because of their small size and lifespan. The mouse lemur has high rates of cognitive decline associated with plaque formation and neurodegeneration including loss of cholinergic neurons in old age [165,168,199,200,201,202,203]. They live 8–14 years in captivity, but are considered elderly after five [204]. The common marmoset *(Callithrix jacchus)* with a lifespan of 7–17 years, is another smaller primate that has shown promise as a monkey model of AD [168,169,205,206]. Perhaps surprisingly, most primates studied so far have shown a higher level of Aβ_1–40_ in plaques than Aβ_1-42_, whereas the common marmoset has a higher level of Aβ_1-42_, similar to human [207].

There is some debate about the usefulness of monkeys as natural models of AD due to the time taken to reach old age and the sporadic nature of naturally occurring AD. At present, monkeys are more often used in AD research for toxicology screening [208], screening of brain imaging compounds [209,210, or for testing of biomarker efficacy [211]. Some ongoing research involves the seeding of amyloid beta or tau in the monkey brain to investigate the proposed spread via a prion-like mechanism [212,213]. Seeding amyloid beta substantially increases the level of amyloid in the marmoset [214], rhesus macaque, and cynomolgus macaques [215], with the latter developing tau pathology and neurodegeneration [216]. This provides a useful tool to investigate the mechanism via which amyloid dysfunction leads to tau dysfunction. TAU injected/seeded rhesus monkeys have also exhibited neurodegeneration after three months [217]. While these models have utility in showing how the disease progresses, they do not capture the underlying mechanism that initiates AD.

The introduction of FAD mutations could solve this problem. Very recently, genetically modified monkey models have begun to be reported. A transgenic cynomolgus monkey model was made by introducing h*APP* with the Swedish, Arctic, and Iberian mutations under a CAG promoter. Plasma Aβ_1–40_ levels were double that of wild type monkeys at birth, while Aβ_1__–__42_ levels were increased 50-fold, increasing the ratio of Aβ_1__–__42_ to Aβ_1–40_ approximately 20-fold [218]. As this is a TG rather than a KI model, the expression of mutant APP is not tissue specific, which may complicate interpretation. Nonetheless, it is a promising development. Around the same time, a KI marmoset model of AD was reported in bioRxiv, carrying the *PSEN1* delta E9 mutation [219]. The ratio of Aβ_1__–__42_ to Aβ_1__–__40_ production in fibroblasts was double that of the controls in the juvenile monkeys, indicating an early pathological change. Both of these potential monkey models are still juveniles, so time will be needed to see what phenotypes arise. However, these are the first reports on the genetically modified monkey model of AD, and they represent exciting developments for the field.

### 5.2. Larger Non-Primate Mammalian Models

Larger mammals outside the primate group present a compromise between the limits of small animal models and the difficulties of working with primates. While primate models have great potential, they are also very expensive and most laboratories do not have the required facilities to house and maintain large numbers for experimental trials. The two most commonly suggested groups are larger companion animals and farm animals. They have a lifespan that is typically 10–15 years and have the advantage of a larger body and more human-like brain than a mouse.

#### 5.2.1. Larger Companion Animals

The domestic dog has been suggested a number of times as a suitable model of AD, both in natural and genetically modified form [220,221,222,223]. Aged dogs develop a dementia-like syndrome called canine cognitive dysfunction (CCD), which has been suggested to be the canine counterpart to AD [180,224,225]. The presence of CCD symptoms has been in more than a quarter of dogs in the 11–12 age range, and nearly 70% in dogs 15–16 [226]. Dogs are the only animal outside humans where cognitive impairment in older age has been reasonably well characterised, and at least three standardised tests exist for assessing CCD [227,228,229]. Dogs naturally develop plaque pathology and cerebral amyloid angiopathy (CAA) [179,230], although it is as still unclear whether this correlates with the symptoms of cognitive decline [178,181,231]. Tau dysfunction and tangles have been reported and associated with cognitive decline [179,182,183]. Aged dogs have occasionally been used as a natural model of AD for therapeutic testing, with aged beagles being used to test the effects of the statin drug Atorvastatin on physiological and behavioural measures of cognitive decline. The research revealed a positive effect on a number of markers for enhanced cognitive function such as biliverdin reductase-A, heme oxygenase-1, and nitric oxide synthase in the brain as well as being significantly correlated with lower discrimination learning error scores [232,233,234]. An immunotherapy effective in mice was once trialled in dogs, with positive results [235].

Domestic cats also develop plaques, tangles, and brain atrophy along with cognitive decline with age [184,236,237]. Research into age-related cognitive dysfunction in cats is not as well developed as that of dogs, but there is increasing interest in this area [185,238,239,240].

#### 5.2.2. Farm Animals

Mammalian farm animals have significant advantages over the aforementioned models in terms of cost and maintenance. Well refined animal husbandry and accelerated reproductive methods means that farm animals can be kept in large numbers at low cost. They can be kept in large groups outdoors, enabling low stress, and natural behaviour. In particular cows, pigs, goats and sheep have been suggested as AD models [144,145,173]. TAU pathology including tangles develop in aged sheep and goats [173,174,175], and plaques have recently been identified in sheep [145]. Plaque and tangle like pathology has also been seen after traumatic brain injury (TBI) in pigs [173]. Pigs and sheep are already in use as models of neurodegenerative disorders, so these will be covered here.

#### 5.2.3. Pigs

With a high degree of genetic similarity, brain structure, and weight, pigs have been selected as a model system for a number of human disorders so there is a growing body of resources for working with this species (see reviews [241,242,243,244]).

Two TG pig models of AD has been reported using minipigs. The first reported model carries a h*APP* transgene with the Swedish mutation driven by the human BDGFβ promoter, resulting in high levels of brain-specific expression [245]. A subsequent publication reported that the animals did not have memory deficits [246]. The most recent report identified the altered activity of *APP* and TAU in astrocytes derived from embryonic stem cells isolated from these TG pigs [247]. No behavioural phenotype has yet been reported.

The second minipig model was reported in 2016. This model carries three copies of a transgene expressing the 695 variant of h*APP* with the Swedish mutation, and a human *PSEN1* transgene with the M146L mutation. Both transgenes were expressed in the brain with normal processing of their protein products. Intraneuronal accumulation of Aβ_1__–__42_ was detected in two pigs: one at 10 months, and one at 18 months [248]. This may represent the early stages of AD, so it will be interesting to see if a more overt pathology is reported in future. Given the rapid development in targeted gene editing technology since these models were made, it should be possible to generate pig models utilising KI methods in the future to enhance construct validity.

#### 5.2.4. Sheep

As above-mentioned, sheep appear to develop plaques and tangles as a natural part of the ageing process [145]. There are many anecdotal reports of age-related cognitive decline in sheep, although this has not been researched and the rate of naturally occurring AD-like dementia is unknown. However, Batten’s disease, a neurodegenerative disease of childhood, has been extensively investigated in sheep due to four models based on naturally occurring genetic mutations [249,250,251,252].

Our lab group has successfully made a transgenic model of the neurodegenerative disorder Huntington’s disease (HD) in sheep [253]. These sheep show early pathological markers of HD, and were used to show urea dysfunction in HD, opening up new avenues of research that are ongoing [254,255]. This line is also being used to test potential therapies [256]. Due to the existence of these sheep models, a number of genetic, physiological and behavioural tools are becoming available. Importantly, the sheep genome has recently been published and annotated [257], and thus the genome of the sheep can now be precisely manipulated for human disease research. In addition, JIVET (juvenile in vitro embryo transfer) technology developed specifically for sheep means that genetically modified ewe lambs can produce viable oocytes at six weeks of age [253]. These oocytes can be fertilized in vitro and implanted into adult recipient ewes, drastically shortening generation times. This can potentially reduce the total time from the implantation of the edited founder embryo, to its offspring being born, to less than one year. This can result in a flock large enough for research use.

Despite their reputation, sheep are reasonably intelligent, and have face recognition systems comparable to humans [258]. This higher cognitive ability makes sheep readily trainable for tests of cognitive function [259]. As in pigs, brain activity in sheep can be monitored longitudinally using EEG [260] and MRI [261]. Our lab group has shown that wild-type sheep amyloid is processed in the same manner as humans, with comparable levels of the disease-related Aβ_1__–__40_ and Aβ_1__–__42_ forms in cerebrospinal fluid (CSF). Similar levels of CSF total-Tau have been found, suggesting that the CSF profile of sheep could be an indicator of disease state.

## 6. Conclusions

Understandably, in a condition defined by pathological features, considerable effort has been expended on deriving models that represent the definition of the disease. This, combined with the time imperative to develop models presenting with plaques and tangles, has resulted in murine models that are a phenocopy of end stage disease but potentially do not represent the natural disease process in humans. Unarguably, the murine models of AD have greatly added to our mechanistic understanding of AD, but they have potentially reached the limits of their utility. There have been widespread calls for more valid models of AD over the last several years, and this has prompted a number of lab groups to turn to large animals. There are a number of potentially suitable large mammals, which can be broadly split into non-human primates and larger companion or farm animals. Primates’ close evolutionary relationship to humans means that discoveries based on them should have high translatability. However the cost and practicalities including breeding and housing sufficient numbers considerably limits their utility, especially for pharmaceutical testing. Larger companion or farm animal models represent a trade-off between primates with their high similarity to humans, and rodents with their ease of use in a laboratory setting. There is considerable potential, especially in farm animals, to produce models that have both high face and construct validity. Regardless, it is clear that to produce an effective treatment for AD, and to avoid the expensive failures at late stage human testing, new preclinical pharmaceutical testing models are required.

## Figures and Tables

**Table 1:** Selected key mouse models of AD and their major phenotypes.

Name	Type of Modification	FAD Mutations	MAPT Mutations	Plaques	Tangles	Neurodegeneration	Reference
PDAPP	Transgenesis	Indiana in *APP*		X			[48]
Tg2576	Transgenesis	Swedish in *APP*		X			[49]
TgCRND8	Transgenesis	Swedish and Indiana in *APP*		X			[50]
PSAPP	Transgenesis	Swedish in *APP*, M146L in *PSEN1*		X			[51]
BRI-Aβ40	Transgenesis	Aβ_1__–__40_ peptide					[52]
BRI-Aβ42	Transgenesis	Aβ_1__–__42_ peptide		X			[52]
5XFAD	Transgenesis	Swedish, Florida, London in *APP*. M146L and L286V in *PSEN1*		X		X	[53]
JNPL3	Transgenesis		P301L in MAPT		X	X	[54]
rTg4510	Transgenesis		P301L in MAPT		X	X	[55]
3xTg	Transgenesis	Swedish in *APP*, M146L in *PSEN1*	P301L in MAPT	X	X	X	[56]
TAPP	Transgenesis	Swedish in *APP*	P301L in MAPT	X	X	X	[57]

**Table 2:** A summary of the large animals in which plaque, tangle pathology, or neurodegeneration with older age has been identified.

Species	Scientific Name	Plaques	Tangles	Neurodegeneration	References
Chimpanzee	*Pan troglodytes*	X	X		[147,148]
Orang-Utan	*Pongo* spp.	X			[149]
Western Gorilla	*Gorilla*	X	X		[150,151]
Eastern Gorilla	*Gorilla beringei*	X	X		[152]
Cynomolgus Monkey	*Macaca fascicularis*	X	X		[153,154,155,156]
Rhesus Macaque	*Macaca mulattas*	X	X		[157,158]
Stump Tailed macaque	*Macaca arctoides*	X	X		[159]
Vervet Monkey	*Chlorocebus aethiops*	X	X		[160]
Baboon	*Papio hamadryas*	X	X		[161,162,163]
Cotton Topped Tamarin	*Saguinus oedipus*	X			[164]
Mouse Lemur	*Microcebus murinus*	X	X	X	[165,166,167]
Common Marmoset	*Callithrix jacchus*	X	X		[168,169]
Squirrel Monkey	*Saimiri sciureus*	X			[170,171]
Pigs	*Sus domesticus*	X *	X *		[172]
Domestic Sheep	*Ovis aries*	X	X		[145,173,174,175]
Domestic Goat	*Capra hircus*		X		[173]
Bactrian Camel	*Camelus bactrianus*	X			[176]
Reindeer	*Rangifer tarandus*		X		[177]
American Bison	*Bison*		X		[177]
Domestic Dog	*Canis familiaris*	X	X	X	[178,179,180,181,182,183]
Domestic Cat	*Felis catus*	X	X	X	[184,185]
Leopard Cat	*Prionailurus bengalensis*	X	X		[186]
Polar Bear	*Ursus maritimus*	X			[187]
Brown Bear	*Ursus arctos*		X		[187]
Black Bear	*Ursus americanus*	X			[188]
Wolverine	*Gulo*	X	X		[189]
Harbor Seal species	*Phoca largha, Phoca vitulina*	X	X		[190]
Sea Lion species	*Eumetopias jubatus, Zalophus californianus, Neophoca cinerea*	X	X		[190]
Walrus	*Odobenus rosmarus*	X	X		[190]

* Found after traumatic brain injury.

## Data Availability

Not applicable.
